# CMR assessment of myocardial mechanics and tissue characterization in patients treated with Anthracycline chemotherapy for acute myeloid leukaemia

**DOI:** 10.1186/1532-429X-14-S1-P182

**Published:** 2012-02-01

**Authors:** Christopher A Miller, Rahul Potluri, Matthias Schmitt

**Affiliations:** 1 University Hospital of South Manchester, Manchester, UK; 2 University of Manchester, Manchester, UK

## Background

Anthracycline-associated cardiomyopathy is a progressive, dose-dependent complication of Anthracycline chemotherapy. The period between Anthracycline therapy and onset of overt heart failure is often years, even decades, and as such Anthracyclines appear to initiate a myocardial injury that remains clinically silent for a substantial period of time. Left ventricular (LV) ejection fraction (EF) is often preserved during this latent period. A more sensitive marker of Anthracycline-associated myocardial injury would allow earlier diagnosis, hence potentially earlier initiation of cardioprotective therapy, and better prognostication.

We assessed the relationship between cumulative Anthracycline dose and myocardial function (global and regional) and myocardial fibrosis (focal and diffuse) in patients who had previously received Anthracycline chemotherapy for acute myeloid leukaemia (AML) with normal or near normal LV EF.

## Methods

15 patients with a prior history of AML underwent 1.5T CMR (Avanto, Siemens). LV volumetric analysis was performed on SSFP images. Mitral inflow was assessed using phase-contrast velocity mapping. Spatial modulation of magnetization was performed on a mid-ventricular short-axis slice in order to assess peak systolic circumferential strain (εcc). T1 mapping was performed pre- and 10-minutes post 0.15mmol/kg gadolinium-DTPA using a modified look locker inversion recovery sequence. ΔR1 ratio [(1/T1myocardium post-contrast) - (1/T1myocardium pre-contrast)] / [(1/T1blood post-contrast) - (1/T1blood pre-contrast)] was used as a marker of diffuse myocardial fibrosis. Late gadolinium enhancement (LGE) images were acquired immediately after post-contrast T1 mapping.

## Results

8 patients were male, mean age 49±17 years. Mean time since AML diagnosis 7±4 years. Mean cumulative Anthracycline dose 200±82mg/m2 (range 75-362). Mean body surface area-indexed end-diastolic volume (EDV) was 71±10mls/m2, mean indexed end-systolic volume (ESV) was 30±5mls/m2 and mean EF was 58±6% (range 51-70%).

There was a significant correlation between cumulative Anthracycline dose and EDV (correlation coefficient 0.43, p=0.03) and ESV (0.43, p=0.03), but no correlation with EF. There were significant correlations between age and ΔR1 ratio (-0.57, p=0.04), age and εcc (0.59, p=0.02) and age and E/A ratio (-0.43, p=0.02). There were also significant correlations between ΔR1 ratio and E/A ratio (0.58, p=0.003), ΔR1 ratio and indexed EDV (0.44, p=0.02) and ΔR1 ratio and indexed ESV (0.41, p=0.04). However, there were no significant correlations between total Anthracycline dose and ΔR1 ratio (0.25, p=0.19), total Anthracycline dose and εcc (0.14, p=0.49) or total Anthracycline dose and E/A ratio (-0.04, p=0.84). No patient had focal LGE.

## Conclusions

In this small group of patients who received chemotherapy for AML a mean of 7 years previously, there was no association between cumulative Anthracycline dose and peak systolic circumferential strain or a marker of diffuse myocardial fibrosis.

## Funding

Dr Miller was supported by a Doctoral Research Fellowship from the National Institute for Health Research, UK (NIHR-DRF-2010-03-98). Dr Schmitt was supported by Greater Manchester Comprehensive Local Research Network funding.

